# Treatment gap and help-seeking for postpartum depression in a rural African setting

**DOI:** 10.1186/s12888-016-0892-8

**Published:** 2016-06-10

**Authors:** Telake Azale, Abebaw Fekadu, Charlotte Hanlon

**Affiliations:** Department of Health Education and Behavioral Sciences, University of Gondar, College of Medicine and Health Sciences, Institute of Public Health, Gondar, Ethiopia; Department of Psychiatry, Addis Ababa University, College of Health Sciences, Addis Ababa, Ethiopia; Department of Psychological Medicine, Centre for Affective Disorders, King’s College London, Institute of Psychiatry, Psychology and Neuroscience, London, UK; King’s College London, Institute of Psychiatry, Psychology and Neuroscience, Centre for Global Mental Health, London, UK

## Abstract

**Background:**

Postpartum depression (PPD) affects more than one in ten women and is associated with adverse consequences for mother, child and family. Integrating mental health care into maternal health care platforms is proposed as a means of improving access to effective care and reducing the ‘treatment gap’ in low- and middle-income countries. This study aimed to determine the proportion of women with PPD who sought help form a health facility and the associated factors.

**Methods:**

A community based, cross-sectional survey was conducted in southern Ethiopia. A total of 3147 women who were between one and 12 months postpartum were screened for depressive symptoms in their home using a culturally validated version of the Patient Health Questionnaire (PHQ-9). Women scoring five or more (indicating potential depressive disorder) (*n* = 385) were interviewed regarding help-seeking behavior. Multiple logistic regression was used to identify factors associated independently with help-seeking from health services.

**Results:**

Only 4.2 % of women (*n* = 16) with high PPD symptoms had obtained mental health care and only 12.7 % of women (*n* = 49) had been in contact with any health service since the onset of their symptoms. In the multivariable analysis, urban residence, adjusted odds ratio (aOR): 4.39 (95 % confidence interval (CI) 1.23, 15.68); strong social support, aOR: 2.44 (95 % CI 1.30, 4.56); perceived physical cause, aOR: 6.61 (95 % CI 1.76, 24.80); perceived higher severity aOR: 2.28 (95 % CI 1.41, 5.47); perceived need for treatment aOR: 1.46 (95 % CI 1.57, 18.99); PHQ score, aOR: 1.14 (95 % CI 1.04, 1.25); and disability, aOR: 1.06 (95 % CI 1.01, 1.15) were associated significantly with help-seeking from health services.

More than half of the women with high levels of PPD symptoms (*n* = 231; 60.0  %) attributed their symptoms to a psychosocial cause and 269 (69.9 %) perceived a need for treatment. Equal proportions endorsed biomedical treatment and traditional or religious healing as the appropriate intervention.

**Conclusion:**

In the absence of an accessible maternal mental health service the treatment gap was very high. There is a need to create public awareness about PPD, its causes and consequences, and the need for help seeking. However, symptom attributions and help-seeking preferences indicate potential acceptability of interventions located in maternal health care services within primary care.

## Background

Postpartum depression (PPD) is the most prevalent mental disorder in the perinatal period in high-income countries (HICs), affecting around 10 to 15 % of women [1]. The prevalence of PPD appears to be at least as high in low- and middle-income countries (LMICs), affecting an estimated 19.8 %[2], although estimates range from zero to 73.7 % [3, 4]. Children born to women with PPD in LMICs are at higher risk of adverse impacts on health, growth and development [5, 6] but have lower access to evidence-based mental health care [7, 8].

Worldwide, at least 56 % of people with non-postpartum depression are estimated to remain untreated [9, 10]. Half of depressed individuals do not seek professional help and up to two-thirds remain untreated or receive inappropriate or inadequate treatment [11]. For PPD the treatment gap appears to be higher than for non-postpartum depression, with studies in HICs indicating that less than a quarter of women with PPD obtain professional help [12, 13]. Exploratory studies have indicated that social norms about good mothering, practical difficulties in accessing care with a young baby and low recognition of symptoms by the woman, her family and healthcare providers may act as specific barriers to accessing mental health care in the post-partum period in HICs [14–16]. The treatment gap for PPD has been little-investigated in LMICs but is expected to be higher than that found in HICs.

In response to this high treatment gap, there has been a global initiative to expand mental health care for perinatal women by integrating interventions into existing maternal health care [17, 18]. Stepped care models of mental health care and contextualized psychosocial interventions have been demonstrated to be feasible, acceptable and effective in the treatment of PPD in LMICs [19, 20], although most studies have been conducted in middle-income rather than low-income countries. However, further studies are needed to understand how these interventions can be scaled up in the highly diverse settings that exist in LMICs. To be successful, scale-up initiatives need to be mindful of the existing help-seeking behaviors and preferences of perinatal women with mental health problems. Understanding current help-seeking may give vital information about existing resources which can be integrated into any planned service; for example, about the potential for collaboration with traditional and faith healers [21].

Help-seeking for postpartum depression has not been studied quantitatively in LMICs. Qualitative exploration indicates that sociocultural factors [22] and causal attributions such as economic difficulty and poor marital relations [23] may be barriers to help-seeking for PPD. In Ethiopia, the period of postpartum confinement, which ranges from 40 days to three months, may be a barrier to help-seeking for women experiencing mental distress [24]. In a previous qualitative study from rural Ethiopia key informants expressed the view that women would be exposed to attack by evil spirit and shamed if they left their home during the postpartum period [25]. In the same study, attribution of mental distress to an exacerbation of preexisting problems, such as poverty and marital discord, rather than to an illness indicated a further barrier to help-seeking from formal health care services [25].

To the best of our knowledge, there is no published study of the treatment gap and help seeking behavior for PPD conducted in a representative population sample in a LMIC setting. Therefore, this study aimed to (1) determine the proportion of women who obtained mental health care for symptoms of PPD, and (2) identify help-seeking behavior and preferences for source of help to inform development of a socioculturally acceptable service intervention for women with PPD.

## Methods

**Study design**: a population-based cross-sectional survey.

**Study area**: the study was carried out in Sodo district, Gurage Zone, Southern Nations, Nationalities and Peoples Region (SNNPR) of Ethiopia. Sodo is located about 100 km south of the capital city, Addis Ababa. In the most recent census, the population was estimated to be 161,952 persons (79,356 men; 82,596 women), with 88 % of the population residing in rural areas [26]. Amharic is the official language in the district, but the second language for the majority of inhabitants. Within Sodo district, there are eight primary care health centers, each linked to health posts which are staffed by community-based health extension workers.

The nearest psychiatric out-patient service is located in Butajira town, 30 km away from the capital of the Sodo district. At the time of the study there were no specialist mental health professionals located within the district and no health care personnel trained in mental health care. However, as part of the Program for Improving Mental health carE (PRIME), plans were being made to integrate mental health care into primary care and maternal health care settings across the district [27]. PRIME is a multi-country project involving five LMICs (Ethiopia, India, Nepal, South Africa and Uganda). PRIME aims to generate evidence on the best approaches for the integration of mental health care into the existing primary and maternal health care services. This study was conducted to inform possible models of intervention for maternal mental health care within the PRIME service model.

### Sampling

As part of PRIME, a census of all households in the district was conducted. However, only 1427 infants (aged less than one year) were recorded within the census, a figure which was much lower than the estimated population from the Central Statistics Agency [28]. Second, we used the immunization report for under one year children from the district health office. Third, we checked the registry of pregnant and postpartum women which is compiled and maintained by community-based health extension workers. Finally, the data collectors identified eligible women in a house-to-house search. These combined approaches resulted in the identification of 3147 women between one and 12 months postpartum. All the identified women were screened using the Patient Health Questionnaire, 9-item depression scale (PHQ-9) [29] and women scoring five or more on the PHQ-9 formed the sample for the study presented in this paper.

### Measures

Postpartum depressive symptoms were measured using the PHQ-9. The PHQ-9 was developed originally to measure depression in primary care settings [30]. The PHQ-9 has been culturally validated for use in several African country settings [31–35] including in postpartum women in rural Ghana [36] and in the primary health care context in rural Ethiopia [37]. In the latter Ethiopian study of the criterion validity of the PHQ-9, a score of five or more was found to have a sensitivity of 83 % and specificity of 75 % for the detection of major depressive disorder.

### Help-seeking behavior

This was assessed using the General Help Seeking Questionnaire (GHSQ) [38]. The GHSQ is a 9-item instrument that was developed to assess future intentions to seek help from a list of culturally-relevant sources. We adapted the GHSQ to ask about actual (rather than intended) behavior and collapsed the response categories to yes/no. The sources of help included in the GHSQ are classified into two major classes: formal and informal. Formal help-seeking includes visiting health professionals (e.g., psychiatrists, psychologists, general practitioners, nurses, etc) or traditional and faith healers [38]. In a rural Ethiopian context, this was adapted to be ‘general health worker’ or ‘mental health worker’. Informal help-seeking is defined as talking about one’s symptoms with parents, friends, a partner or other relative.

The woman’s explanatory model of postpartum depressive symptoms was investigated using the Short Explanatory Model Interview (SEMI) [39]. The SEMI is a semi-structured questionnaire with open ended questions to be documented verbatim and coded using a contextualized set of possible categories. A version of SEMI was adapted for Ethiopia with an expert consensus meeting involving mental health professionals and qualitative researchers with experience working in the study site. The women’s perceptions of causes, severity, treatment needs and options for symptoms of postpartum depression were assessed.

Disability was measured using the World Health Organization Disability Assessment Tool (WHODAS) [40] which covers the functional domains of understanding and communicating, getting around, self-care, getting along with people, life activities, and participation in society. Each item was scored from 1 (none) to 5 (extreme or cannot do), with the total WHODAS score ranging from 36 to 180. The WHODAS has been used in Ethiopia in perinatal women in the neighboring district and found to have convergent validity and acceptability [41].

Social support was measured using the Oslo Social Support Scale (OSSS-3) [42] The OSSS-3 total score ranges between three and 14. Scores from 3 to 8 are considered to indicate poor support, scores from 9 to 11 indicate intermediate support, and a score between 12 and 14 is considered to indicate strong social support. Although these cut-off points have not been validated in the Ethiopian context, the OSSS-3 categories were used in a community study in the same Ethiopian district and showed good utility [43].

The Barriers to Access to Care Evaluation (BACE) was adapted for use in the study site [44]. Twenty-three out of 30 original items were used in this study as some of the items were not applicable for women with PPD living in a rural African context. For example items like “*Concern that it might harm my chances when applying for jobs*” and “*Not wanting a mental health problem to be on my medical records”* were excluded. The tool asks about a range of issues that have ever stopped, delayed or discouraged an individual from getting, or continuing with, professional care for a mental health problem on a scale from 0 (not at all) to 3 (a lot). The domains of potential barriers include individual perception (including stigma), infrastructure, knowledge, social support, attitude of respondents towards the available treatment and previous experiences.

### Data collection and quality assurance

Women were interviewed in their homes privately by 36 trained data collectors who were trained for 9 days. The data collectors were recruited from the district and the sub-districts or, if no eligible person was available, applicants from the neighboring sub-districts were recruited. The educational levels of the data collectors ranged from tenth grade completed to first degree. They were supervised by four supervisors who were also trained and assisted by the investigators. The supervisors were diploma or degree graduates. The data collectors went house-to-house, explained the purpose of the research and either gave an information sheet to the woman or read the information for those who were unable to read. Women who consented to participate were interviewed at a time convenient for them within a day or two of initial contact. A pre-test was conducted in three sub-districts near the study area. Data were collected between April and June 2014.

### Data management and analysis

Data were double entered into EpiData version 3.1 and exported to SPSS-20 for analysis. Frequencies, percentages, and mean values were used to describe the categorical and continuous variables. Bivariate analyses were carried out to investigate the association between help-seeking behavior of women with symptoms of PPD from a general health facility and each of the independent variables. This outcome was of interest because of the plan to integrate mental health care for women into general health services. All variables with a *p*-value <0.2 were included in the multivariable model. Adjusted odds ratios with associated 95 % confidence intervals were reported in the final multiple logistic regression model.

## Results

A total of 385 women with a PHQ-9 score of five or more were included in the analyses. Only one woman was excluded and referred for specialist mental health care after she was found to have probable psychotic symptoms. No women refused to participate in the study.

The prevalence of depression across the postpartum months was 12.7, 11.2, and 12.5 % in the months one to three, four to six, and seven or more, respectively.

### Socio-demographic characteristics

The mean PHQ score of the participants was 7.8 (Standard Deviation (SD) 3.0). The mean age of the respondents was 28.8 years (SD 5.2) years). Almost all women were married (97.1 %; *n* = 374) and rural residents (94.3 %; *n* = 363). More than two-thirds, (70.4 %; *n* = 271) were unable to read and write. The vast majority (94.8 %; *n* = 365) were followers of Orthodox Christianity and 91.7 % (*n* = 353) were Gurage by ethnicity (See Table 1).

**Table 1:** Socio-demographic characteristics of women with symptoms of postpartum depression in Sodo district, Ethiopia, 2014 (*n* = 385)

Socio-demographic characteristic	Frequency	Percent
Age in years		
Less than 20	28	7.3
20–29	174	45.2
30–39	175	45.5
40 or above	8	2.1
Marital status		
Married	374	97.1
Single/widowed/divorced	11	2.9
Residence		
Rural	363	94.3
Urban	22	5.7
Education		
No formal education	308	80.0
Formal education	77	20
Occupation		
House wife	317	82.3
Private (shop, other trade)	49	12.8
No job	19	4.9
Religion		
Orthodox	365	94.8
Protestant	12	3.1
Muslim	6	1.6
Other	2	0.6
Ethnicity		
Gurage	353	91.7
Oromo	26	6.8
Amhara	5	1.3
Other	1	0.3

Half (50.9 %; *n* = 196) of the women were grandmultiparious. Just less than half (44.4 %; *n* = 171) of women perceived that their wealth was lower than their neighbors. More than a quarter, (27 %; *n* = 104) perceived that they had poor social support. For the married women, about 80 % reported having a good relationship with their husband. However, 18.2 % said that their husbands did not live with them, for example, because of working away from home (12.5 %) or due to having another wife (3.4 %). Nearly a quarter of women (24.7 %; *n* = 95) reported that their husbands drank too much alcohol.

Mild to extreme impairment was reported by 339 (88 %) of women in one or more of the six domains of functioning. However, only 2.6 % reported that they had been unable to function for more than 15 days in the last one month due to disability.

### Help-seeking from general health care facilities

Only 50 women (12.9 %) had ever had contact with health services following the onset of symptoms of PPD. Only 4.2 % (*n* = 16) reported that they accessed mental health care. From the informal sources of help, husbands were the most frequently sought source of help (*n* = 236; 61.3 %). The least frequently sought source of help was a sorcerer (*n* = 12; 3.1 %) (See Table 2). Out of 276 (71.6 %) women who said that their illness needed treatment, 192 (49.9 %) endorsed modern medicine as their preferred type of treatment.

**Table 2:** Frequency distribution of items of the General Help-seeking Questionnaire (GHSQ) among women with symptoms of postpartum depression (*n* = 385)

Sources of help	Number	Percent
Formal		
General health professional (any)		
Yes	49	12.7
No	336	87.3
Mental health professional		
Yes	16	4.2
No	369	95.8
Religious leader		
Yes	45	11.7
No	340	88.3
Traditional healer		
Yes	20	5.2
No	365	94.8
Sorcerer/witch doctor		
Yes	12	3.1
No	373	96.9
Informal		
Husband		
Yes	236	61.3
No	149	38.7
Friends		
Yes	182	47.3
No	203	52.7
Parents		
Yes	180	46.8
No	205	53.2
Other relatives		
Yes	140	36.4
No	245	63.6

The perceived cause for 231 (60.0 %) of respondents was psychosocial, which included financial difficulty, an unsupportive partner and “thinking too much”. The most frequently mentioned factors that helped PPD symptoms get better were performing religious activities (prayer, using holy water and attending church), discussing with significant others, or thinking less about the problem and being relaxed (be calm, sleep, take rest). The factors that were mentioned as aggravating PPD symptoms were work overload, psychological (persistent worry, thinking too much, upset), and interpersonal conflict. About two-thirds of women (62.1 %; *n* = 239) attributed their problem to internal events such as their failure to do something that they should have done or doing something they should not have done.

**Fig. 1:**
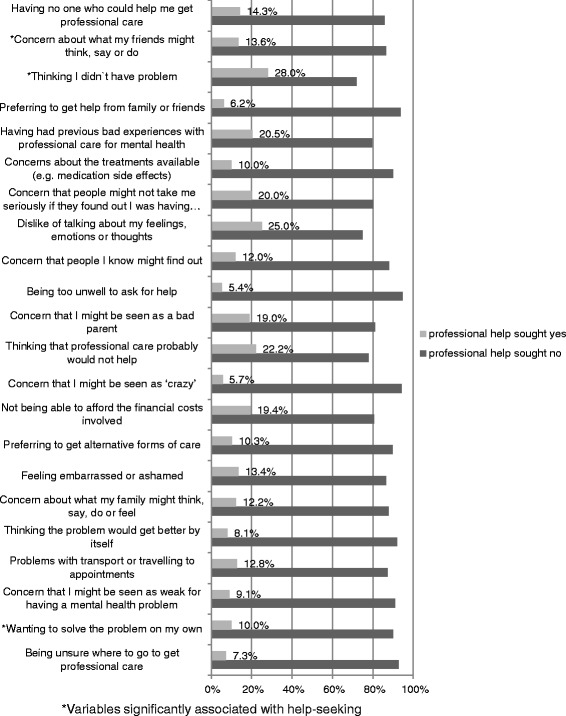
The frequency of the 23 items of the Barriers to Access to Care Evaluation

Thinking the problem would get better by itself (76.1 %; *n* = 293); the woman wanting to solve the problem by herself (66.7 %; *n* = 257); fear of cost (56.0 %; *n* = 216); and distance (50.4 %; *n* = 194) were identified as the most common barriers to accessing care. (See Figure 1).

In the multivariable model, urban residence, adjusted Odds Ratio (aOR): 3.76, 95 % CI (1.07, 13.12); multiparity, aOR: 1.16, 95 % CI (1.01, 1.33); perceived physical cause, aOR: 6.84, 95 % CI (2.06, 22.75); perceived severity, aOR: 3.10, 95 % CI (1.13, 8.48); perceived need for treatment, aOR: 6.24, 95 %CI (1.82, 21.35); PHQ score, aOR: 1.13, 95 % CI (1.04, 1.24) for every one point increase; and overall disability score on the WHODAS, aOR: 1.01, 95 % CI (1.00, 1.03) were found to be statistically significantly associated with help seeking behavior from a health facility.

The following self-reported barriers to care were associated with not seeking help from health services in the multivariable model: wanting to solve the problem by herself aOR: 0.51, 95 % CI (0.30, 0.88) and thinking that they did not have a problem aOR: 0.36, 95 % CI (0.18, 0.71). Unexpectedly, concern about what friends may think or say was associated with increased help-seeking from health services: aOR: 2.45, 95 % CI (1.21, 4.96) (Table 3).

**Table 3:** Factors associated with help-seeking behavior from a health facility for postpartum depression symptoms at Sodo district, 2014 (*n* = 385)

Characteristics	Help-seeking from health facility		Crude odds ratio (95 % confidence interval)	AOR(95 % CI)
	Yes N (%)	No N (%)		
Socio-demographic factors				
Age in years (mean = 28.8, SD = 5.23)			1.03 (0.97,1.09)	0.96 (0.88, 1.04)
*Residence*				
Rural	42 (11.6)	321 (88.4)	1	1
Urban	7 (31.8)	15 (68.2)	3.56 (1.37, 9.25)	4.39 (1.23, 15.68)
*Education*				
No formal education	38 (12.3)	270 (87.7)	1	1
Formal education	11 (14.3)	66 (85.7)	0.84 (0.41, 1.74)	0.97 (0.37, 2.52)
Experienced hunger				
Yes	19 (15.1)	107 (84.9)	1.35 (0.73, 2.51)	0.70 (0.30, 1.67)
No	30 (11.6)	229 (88.4)	1	1
Relative wealth				
Lower	22 (12.9)	149 (87.1)	1	1
Same or better	27 (19.3)	187 (80.7)	1.02 (0.56, 1.86)	0.56 (0.05, 5.36)
Social support				
Good	34 (28.8)	247 (71.2)	2.65 (1.38, 5.11)	2.44 (1.30, 4.56)
Poor	15 (14.4)	89 (85.6)	1	1
Perceived cause				
Psychosocial	15 (6.5)	216 (93.5)	4.17 (2.02, 8.59)	4.24 (1.94, 9.26)
Physical	20 (22.5)	69 (77.5)	8.64 (2.76, 27.00)	6.61 (1.76, 24.80)
Supernatural	6 (37.5)	10 (62.5)	2.81 (1.19, 7.05)	2.85 (1.06, 7.67)
I don’t know	8 (16.3)	41 (83.7)	1	1
Perceived severity				
Severe	30 (49.3)	123 (50.7)	2.73 (1.47, 5.06)	2.28 (1.41, 5.47)
Not severe	19 (16)	213 (84)	1	1
Perceived need for treatment				
Yes	46 (17.1)	223 (82.9)	1.77 (2.36, 25.53)	1.46 (1.57, 18.99)
No	3 (5.4)	113 (94.6)	1	1
Want to solve problem self	26 (19.9)	231 (80.1)	0.24 (0.09, 0.65)	0.51 (0.30, 0.88)
Professional care wouldn’t help	8 (12.4)	116 (87.6)	0.47 (0.25, 0.89)	0.55 (0.28, 1.09)
Thinking no problem	9 (11.7)	142 (88.3)	0.38 (0.20, 0.72)	0.36 (0.18, 0.71)
Concern about what friends think	17 (42.3)	78 (57.7)	2.90 (1.70, 4.94)	2.45 (1.21, 4.96)
PHQ score			1.13 (1.04, 1.23)	1.14 (1.04, 1.25)
WHODAS Score			1.45 (1.03, 2.05)	1.06 (1.00, 1.15)

## Discussion

In this population-based survey of postpartum women with high levels of depressive symptoms in rural Ethiopia, the treatment gap for biomedical care was very high. Levels of help-seeking from any type of biomedical care provider were low (12.7 %) and only 4.2 % of women (*n* = 15) accessed mental health care. Many women relied on non- professional sources of help such as from their partner, parents and friends. However, the majority (69.9 %) of women reported that they needed professional help for their symptoms, and equal proportions endorsed biomedical health care and traditional or religious healing.

The finding that one in ten women had been in contact with general health services since the onset of PPD symptoms does not indicate that these women would have been looking for, or received, mental health care. At the time of the study there was no mental health service within general health care facilities [45]. Instead, it is likely that somatic symptoms of depression were the motivating factor [46]. This accords with our finding that help-seeking from health services was higher in women who attributed their symptoms to a physical cause. There are no mental health professionals working within the district, and so it is noteworthy that 4.2 % of women sought out mental health care from the psychiatric nurse-led out-patient clinic in the neighboring district (30 to 50 km away). Indicators of severity of PPD (increasing number of depression symptoms, perceived severity and level of functional impairment) were all associated with higher levels of help-seeking from health services. This supports the notion that symptoms were troublesome for women and having an adverse impact upon their lives.

The willingness of 12 % of women to seek help from health services, together with the further 49.8 % who expressed a preference for biomedical care for their problem, indicates potential acceptability for plans to integrate mental health care into maternal health care platforms in this rural Ethiopian district. However, possible barriers to help-seeking from health services were also apparent in our study. Women from rural areas were less likely to seek help from health services, which may reflect a combination of factors, including proximity, level of autonomy and awareness of the potential benefits of treatment. Contrary to expectations, there was no association between indicators of socio-economic status (e.g. experiencing hunger in the last one month, relative wealth) or educational status and help-seeking from health services. The limited variation in socio-economic status in rural communities may have contributed to this negative finding. More than 80 % of the respondents did not receive formal education and are housewives by occupation. In line with many studies, perceived social support was associated with help seeking in this study [47–49]. Urban residence was associated with help seeking and is likely to be due to a better access of urban residents to health institutions and contact with health professionals for routine maternal and child health services. Education and knowledge about depression have been found to be facilitators of help seeking in some studies [50]. However, systematic reviews of help seeking interventions revealed that knowledge tends to improve attitudes but not behavior [51, 52], although education can create access to health service use through better information and income.

Some of the apparent “barriers” to help-seeking, for example, wanting to solve the problem on their own or not framing their symptoms as a problem, may reflect the attribution of PPD symptoms to social adversity, as was found in a qualitative study of PPD conceptualization in the neighboring district [25]. As a screening tool for depressive symptoms was used in this study, which has a relatively low positive predictive value [37], some of the women may be suffering from transient distress which does not meet diagnostic criteria for depression. Therefore caution needs to be exercised not to pathologize social suffering as depressive disorder in this setting. On the other hand, low levels of awareness about mental health conditions and their treatability may be a barrier to women who could otherwise benefit from biomedical treatment. Longitudinal evaluation of the outcomes for women with PPD who limit their help-seeking to friends and family, or who seek formal sector care from traditional and religious healers, is needed in order to understand whether a narrow view of ‘evidence-based care’ overstates the apparent ‘treatment gap’ in this setting. Only by providing evidence of the effectiveness, or otherwise, of the non-biomedical approaches to care for PPD can we fully understand the unmet needs for care; however, it was evident that there was a desire for help beyond that which had already been obtained within the sociocultural context.

The finding that women who endorsed “concern about what my friends might think, say, or do” if they attended a health facility (intended to tap into stigma) were actually significantly more likely to seek help from a health facility was unexpected. It seems probable that women interpreted this question as meaning that expression of concern from friends would increase attendance at a health facility. Most of the participants talked to their husbands and many sought help from parents, friends and other relatives. Those individuals who had discussed their problems with their significant others could be prompted to seek professional help if aware of the availability of care [48].

Among the several barriers to help seeking identified within the study, thinking that professional care wouldn’t help, and thinking that they didn’t have a problem requiring treatment were associated significantly with lower levels of help seeking from health facilities. This indicates low level of awareness about the potential benefits of biomedical treatments for PPD. Increasing mental health literacy within the population, including amongst husbands, may give women more choices about the care they seek. Furthermore, although not significant, fear of cost and distance from mental health care were frequently mentioned as barriers to help-seeking. Integrating maternal mental health care into primary health care services may, therefore, improve the uptake of mental health service.

The perception of women that psychosocial factors are the major contributors of their symptoms and their attribution to internal events might have led in their religious and emotion focused coping strategies that they mentioned as relieving factors of symptoms. This may help for the contextualization of psychosocial intervention that builds on things that women find to be helpful.

### Limitations

The time frame for help seeking was not specified in the questionnaire. Although the participants were asked for the presence of PPD symptoms in the preceding two weeks, the symptoms might have been present for several months, including during pregnancy when most women were likely to have had contact with health professionals for antenatal care, and this could have inflated the estimates of contact with services. Another limitation is that no reliability testing was carried out for the coding of responses to the Short Explanatory Model Interview. A further limitation is that we do not know how women managed to access the existing mental health service in the neighboring district. Although we made strenuous efforts to ensure complete ascertainment of postpartum women, it is possible that we missed some women and this could have reduced the generalizability of the findings.

## Conclusion

Help seeking from health professionals for symptoms of postpartum depression was very low in the study district. In the absence of an accessible maternal mental health service, the treatment gap for PPD was very high. However, symptom attributions and help-seeking preferences indicate potential acceptability of interventions located in maternal health care services within primary care. Creating public awareness about PPD, its causes and consequences, and the need for help seeking are necessary steps to support the integration of mental health into primary care-based maternal health care.

## Abbreviations

BACE, Barriers to Access to Care Evaluation; CSA, Central Statistics Agency; GHSQ, General Help Seeking Questionnaire; HIC, high income country; LMIC, low and middle income country; OSSS, Oslo Social Support Scale; PHQ, patient health questionnaire; PPD, postpartum depression; PRIME, programme for improving mental health care; SEMI, short explanatory model interview; SNNPR, South Nations and Nationalities and Peoples Region; WHODAS, World Health Organization Disability Assessment Schedule
